# Crystal structure of (*E*)-2-[(4-hy­droxy­benzyl­idene)aza­nium­yl]benzoate

**DOI:** 10.1107/S1600536814018273

**Published:** 2014-08-16

**Authors:** M. Nawaz Tahir, Abdul Haleem Khan, Hazoor Ahmad Shad

**Affiliations:** a Department of Physics, University of Sargodha, Sargodha, Pakistan; b Department of Pharmacy Services, Jinnah Hospital, Lahore, Pakistan; c Department of Chemistry, University of Sargodha, Sargodha, Pakistan

**Keywords:** crystal structure, Schiff bases, aza­nium–carboxyl­ate zwitterion, hydrogen bonding

## Abstract

The title Schiff base, C_14_H_11_NO_3_, crystallizes as a zwitterion (*i.e.* proton transfer from the carb­oxy­lic acid group to the imine N atom). The dihedral angle between the aromatic rings is 19.59 (6)° and an intra­molecular N—H⋯O hydrogen bond closes an *S*(6) ring. In the crystal, inversion dimers linked by pairs of O—H⋯O hydrogen bonds generate *R*_2_^4^(24) loops. The dimers are linked by C—H⋯O inter­actions, generating (211) sheets.

## Related literature   

For related structures, see: Hang *et al.* (2010 ▶); Ligtenbarg *et al.* (1999 ▶); Trzesowska-Kruszynska (2010 ▶).
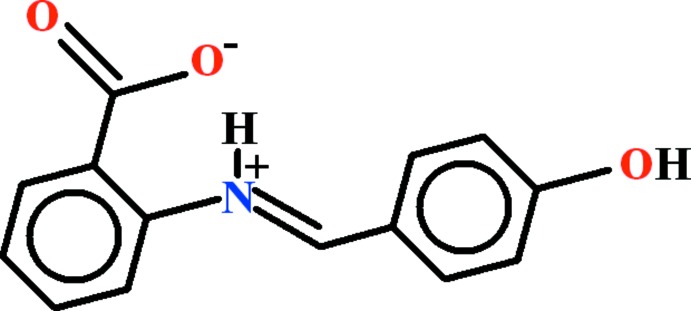


## Experimental   

### Crystal data  

C_14_H_11_NO_3_
*M* *_r_* = 241.24
Monoclinic, 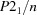

*a* = 3.8612 (5) Å
*b* = 15.280 (3) Å
*c* = 18.604 (3) Å
β = 90.347 (8)°
*V* = 1097.6 (3) Å^3^
*Z* = 4
Mo *K*α radiation
μ = 0.10 mm^−1^
*T* = 296 K
0.38 × 0.17 × 0.15 mm

### Data collection  

Bruker Kappa APEXII CCD diffractometer
Absorption correction: multi-scan (*SADABS*; Bruker, 2007 ▶) *T*_min_ = 0.962, *T*_max_ = 0.985
17364 measured reflections
2089 independent reflections
1363 reflections with *I* > 2σ(*I*)
*R*_int_ = 0.065

### Refinement  

*R*[*F* ^2^ > 2σ(*F* ^2^)] = 0.059
*wR*(*F* ^2^) = 0.124
*S* = 1.15
2089 reflections
169 parameters
H atoms treated by a mixture of independent and constrained refinement
Δρ_max_ = 0.18 e Å^−3^
Δρ_min_ = −0.23 e Å^−3^

### 

Data collection: *APEX2* (Bruker, 2007 ▶); cell refinement: *SAINT* (Bruker, 2007 ▶); data reduction: *SAINT*; program(s) used to solve structure: *SHELXS97* (Sheldrick, 2008 ▶); program(s) used to refine structure: *SHELXL97* (Sheldrick, 2008 ▶); molecular graphics: *ORTEP-3 for Windows* (Farrugia, 2012 ▶) and *PLATON* (Spek, 2009 ▶); software used to prepare material for publication: *WinGX* (Farrugia, 2012 ▶) and *PLATON* (Spek, 2009 ▶).

## Supplementary Material

Crystal structure: contains datablock(s) global, I. DOI: 10.1107/S1600536814018273/hb7268sup1.cif

Structure factors: contains datablock(s) I. DOI: 10.1107/S1600536814018273/hb7268Isup2.hkl

Supporting information file. DOI: 10.1107/S1600536814018273/hb7268Isup3.cml

**Fig. 1.**
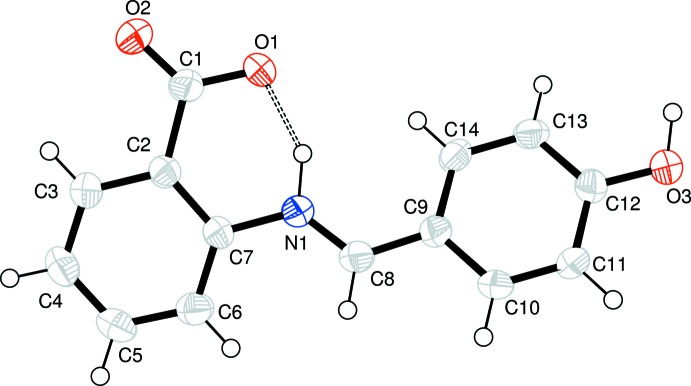
View of the title compound with displacement ellipsoids drawn at the 50% probability level. The dotted line represents the intramolecular H-bond.

. DOI: 10.1107/S1600536814018273/hb7268fig1.tif
View of the title compound with displacement ellipsoids drawn at the 50% probability level. The dotted line represents the intra­molecular H-bond.

**Fig. 2.**
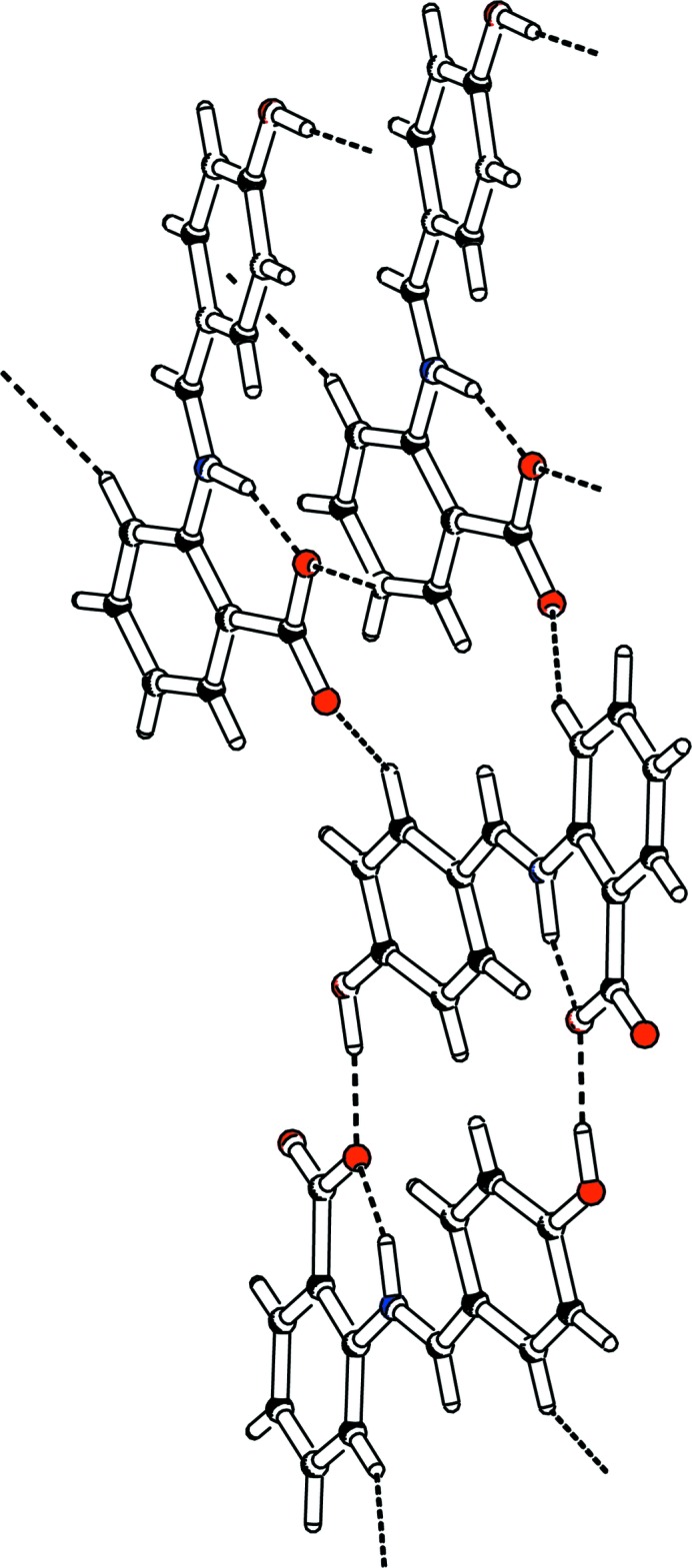
The partial packing, which shows that molecules form dimers which are interlinked.

. DOI: 10.1107/S1600536814018273/hb7268fig2.tif
The partial packing, which shows that mol­ecules form dimers which are inter­linked.

CCDC reference: 1018737

Additional supporting information:  crystallographic information; 3D view; checkCIF report

## Figures and Tables

**Table 1:** Hydrogen-bond geometry (Å, °)

*D*—H⋯*A*	*D*—H	H⋯*A*	*D*⋯*A*	*D*—H⋯*A*
O3—H3*A*⋯O1^i^	0.95 (4)	1.71 (4)	2.656 (3)	173 (3)
N1—H1⋯O1	1.00 (3)	1.62 (3)	2.522 (3)	148 (2)
C6—H6⋯O2^ii^	0.93	2.51	3.397 (4)	160

Symmetry codes: (i) 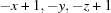; (ii) 
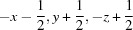
.

## supplementary crystallographic information

### S1. Comment

The title compound (I), (Fig. 1) has been synthesized for forming different metal complexes.

The crystal structures of *N*-(2-Carboxyphenyl)salicylidenimine (Ligtenbarg *et al.*, 1999), 2-((4-(dimethylamino)benzylidene)ammonio) benzoate pentahydrate (Trzesowska-Kruszynska, 2010) and 2-[(2-hydroxy-4-methoxybenzylidene)azaniumyl]benzoate monohydrate (Hang, *et al.*, 2010) have been published which are related to the title compound (I).

The title compound has been crystalized in the zwitterion form. In (I) the moieties of 2-aminobenzoic acid A (C1—C7/N1/O1/O2) and the 4-hydroxybenzalehyde B (C8—C14/O3) are planar with r.m.s. deviation of 0.0133 and 0.0219 Å, respectively. The dihedral angle between A/B is 19.589 (58)°. In (I), *S*(6) ring motif is present due to H-bonding of N—H···O type (Table 1, Fig. 1). The molecules are dimerized from end to end due to H-bondings of O—H···O type (Table 1, Fig. 2) and form *R*_2_^4^(24) loop. The dimers are further interlinked due to C—H···O bonds.

### S2. Experimental

Equimolar quantities of 2-aminobenzoic acid and 4-hydroxybenzaldehyde were refluxed in methanol along with few drops of acetic acid as catalyst for 2 h. The resulting solution was kept at room temperature which afforded yellow needles after two days.

### S3. Refinement

The coordinates of H1 and H3A were refined. The H-atoms were positioned geometrically (C–H = 0.93 Å) and refined as riding with *U*_iso_(H) = *xU*_eq_(C, N, O), where *x* = 1.5 for hydroxy & *x* = 1.2 for other H-atoms.

### Crystal data

**Table:**

C_14_H_11_NO_3_	*F*(000) = 504
*M**_r_* = 241.24	*D*_x_ = 1.460 Mg m^−^^3^
Monoclinic, *P*2_1_/*n*	Mo *K*α radiation, λ = 0.71073 Å
*a* = 3.8612 (5) Å	Cell parameters from 1363 reflections
*b* = 15.280 (3) Å	θ = 1.8–26.0°
*c* = 18.604 (3) Å	µ = 0.10 mm^−^^1^
β = 90.347 (8)°	*T* = 296 K
*V* = 1097.6 (3) Å^3^	Cut needle, yellow
*Z* = 4	0.38 × 0.17 × 0.15 mm

### Data collection

**Table:**

Bruker Kappa APEXII CCD diffractometer	2089 independent reflections
Radiation source: fine-focus sealed tube	1363 reflections with *I* > 2σ(*I*)
Graphite monochromator	*R*_int_ = 0.065
Detector resolution: 8.00 pixels mm^-1^	θ_max_ = 26.0°, θ_min_ = 1.7°
ω scans	*h* = −4→4
Absorption correction: multi-scan (*SADABS*; Bruker, 2007)	*k* = −18→18
*T*_min_ = 0.962, *T*_max_ = 0.985	*l* = −22→22
17364 measured reflections	

### Refinement

**Table:**

Refinement on *F*^2^	Primary atom site location: structure-invariant direct methods
Least-squares matrix: full	Secondary atom site location: difference Fourier map
*R*[*F*^2^ > 2σ(*F*^2^)] = 0.059	Hydrogen site location: inferred from neighbouring sites
*wR*(*F*^2^) = 0.124	H atoms treated by a mixture of independent and constrained refinement
*S* = 1.15	*w* = 1/[σ^2^(*F*_o_^2^) + (0.0187*P*)^2^ + 1.0858*P*] where *P* = (*F*_o_^2^ + 2*F*_c_^2^)/3
2089 reflections	(Δ/σ)_max_ < 0.001
169 parameters	Δρ_max_ = 0.18 e Å^−^^3^
0 restraints	Δρ_min_ = −0.23 e Å^−^^3^

### Special details

**Table:**

Geometry. All e.s.d.'s (except the e.s.d. in the dihedral angle between two l.s. planes) are estimated using the full covariance matrix. The cell e.s.d.'s are taken into account individually in the estimation of e.s.d.'s in distances, angles and torsion angles; correlations between e.s.d.'s in cell parameters are only used when they are defined by crystal symmetry. An approximate (isotropic) treatment of cell e.s.d.'s is used for estimating e.s.d.'s involving l.s. planes.
Refinement. Refinement of *F*^2^ against ALL reflections. The weighted *R*-factor *wR* and goodness of fit *S* are based on *F*^2^, conventional *R*-factors *R* are based on *F*, with *F* set to zero for negative *F*^2^. The threshold expression of *F*^2^ > σ(*F*^2^) is used only for calculating *R*-factors(gt) *etc*. and is not relevant to the choice of reflections for refinement. *R*-factors based on *F*^2^ are statistically about twice as large as those based on *F*, and *R*-factors based on ALL data will be even larger.

### Fractional atomic coordinates and isotropic or equivalent isotropic displacement parameters (Å^2^)

**Table:**

	*x*	*y*	*z*	*U*_iso_*/*U*_eq_	
O1	0.1341 (6)	−0.08844 (13)	0.32981 (10)	0.0469 (6)	
O2	0.0144 (7)	−0.18735 (14)	0.24574 (12)	0.0580 (7)	
O3	0.6380 (6)	0.22716 (14)	0.59807 (11)	0.0489 (6)	
H3A	0.701 (9)	0.177 (2)	0.6251 (19)	0.073*	
N1	−0.0068 (6)	0.07058 (16)	0.30803 (13)	0.0360 (6)	
H1	0.067 (7)	0.016 (2)	0.3337 (15)	0.043*	
C1	0.0114 (8)	−0.1120 (2)	0.26817 (16)	0.0394 (7)	
C2	−0.1414 (7)	−0.04003 (18)	0.22116 (14)	0.0328 (7)	
C3	−0.2813 (8)	−0.0637 (2)	0.15519 (15)	0.0398 (7)	
H3	−0.2817	−0.1223	0.1417	0.048*	
C4	−0.4196 (8)	−0.0021 (2)	0.10915 (16)	0.0460 (8)	
H4	−0.5103	−0.0192	0.0649	0.055*	
C5	−0.4233 (8)	0.0847 (2)	0.12868 (16)	0.0461 (8)	
H5	−0.5171	0.1261	0.0975	0.055*	
C6	−0.2890 (8)	0.1107 (2)	0.19417 (16)	0.0416 (8)	
H6	−0.2931	0.1694	0.2074	0.050*	
C7	−0.1485 (7)	0.04873 (18)	0.23973 (14)	0.0332 (7)	
C8	0.0590 (7)	0.14802 (19)	0.33225 (15)	0.0370 (7)	
H8	0.0125	0.1955	0.3025	0.044*	
C9	0.1993 (7)	0.16514 (18)	0.40216 (15)	0.0342 (7)	
C10	0.2987 (7)	0.25053 (19)	0.41916 (16)	0.0384 (7)	
H10	0.2674	0.2947	0.3854	0.046*	
C11	0.4416 (8)	0.27050 (19)	0.48472 (16)	0.0397 (7)	
H11	0.5063	0.3278	0.4950	0.048*	
C12	0.4901 (8)	0.20511 (19)	0.53595 (15)	0.0365 (7)	
C13	0.3814 (7)	0.12005 (18)	0.52047 (15)	0.0375 (7)	
H13	0.4051	0.0764	0.5550	0.045*	
C14	0.2400 (8)	0.10038 (19)	0.45483 (15)	0.0376 (7)	
H14	0.1700	0.0433	0.4451	0.045*	

### Atomic displacement parameters (Å^2^)

**Table:**

	*U*^11^	*U*^22^	*U*^33^	*U*^12^	*U*^13^	*U*^23^
O1	0.0702 (15)	0.0380 (12)	0.0324 (12)	0.0036 (11)	−0.0144 (11)	0.0019 (9)
O2	0.0942 (19)	0.0323 (12)	0.0474 (14)	0.0034 (13)	−0.0117 (13)	−0.0025 (11)
O3	0.0679 (16)	0.0367 (13)	0.0419 (13)	−0.0001 (11)	−0.0187 (11)	−0.0015 (10)
N1	0.0437 (15)	0.0313 (14)	0.0329 (13)	0.0036 (11)	−0.0026 (11)	−0.0010 (11)
C1	0.0498 (19)	0.0336 (17)	0.0347 (17)	−0.0006 (14)	0.0009 (15)	0.0032 (14)
C2	0.0340 (16)	0.0343 (16)	0.0300 (15)	0.0000 (13)	0.0007 (13)	0.0007 (12)
C3	0.0438 (18)	0.0382 (18)	0.0374 (17)	−0.0019 (14)	−0.0049 (14)	−0.0030 (14)
C4	0.0479 (19)	0.056 (2)	0.0344 (18)	0.0010 (16)	−0.0085 (15)	−0.0005 (15)
C5	0.050 (2)	0.048 (2)	0.0400 (18)	0.0060 (16)	−0.0051 (16)	0.0115 (16)
C6	0.0443 (19)	0.0354 (17)	0.0452 (19)	0.0038 (14)	−0.0015 (15)	0.0032 (14)
C7	0.0370 (17)	0.0347 (17)	0.0278 (15)	−0.0001 (13)	−0.0005 (13)	0.0003 (12)
C8	0.0401 (17)	0.0319 (16)	0.0390 (17)	0.0000 (13)	−0.0009 (14)	0.0033 (14)
C9	0.0350 (16)	0.0313 (16)	0.0362 (16)	0.0019 (13)	−0.0024 (13)	−0.0017 (13)
C10	0.0439 (18)	0.0305 (16)	0.0407 (18)	0.0027 (14)	−0.0066 (14)	0.0064 (13)
C11	0.0461 (18)	0.0271 (16)	0.0457 (19)	−0.0011 (13)	−0.0075 (15)	−0.0018 (14)
C12	0.0389 (17)	0.0359 (17)	0.0345 (16)	0.0020 (13)	−0.0027 (14)	−0.0028 (13)
C13	0.0472 (19)	0.0289 (16)	0.0365 (17)	0.0018 (14)	0.0006 (14)	0.0047 (13)
C14	0.0442 (18)	0.0280 (16)	0.0404 (17)	−0.0022 (13)	0.0000 (14)	−0.0035 (13)

### Geometric parameters (Å, º)

**Table:**

O1—C1	1.289 (3)	C5—H5	0.9300
O2—C1	1.225 (3)	C6—C7	1.380 (4)
O3—C12	1.329 (3)	C6—H6	0.9300
O3—H3A	0.95 (4)	C8—C9	1.430 (4)
N1—C8	1.291 (3)	C8—H8	0.9300
N1—C7	1.420 (3)	C9—C10	1.396 (4)
N1—H1	1.00 (3)	C9—C14	1.401 (4)
C1—C2	1.522 (4)	C10—C11	1.370 (4)
C2—C3	1.386 (4)	C10—H10	0.9300
C2—C7	1.400 (4)	C11—C12	1.393 (4)
C3—C4	1.378 (4)	C11—H11	0.9300
C3—H3	0.9300	C12—C13	1.395 (4)
C4—C5	1.375 (4)	C13—C14	1.368 (4)
C4—H4	0.9300	C13—H13	0.9300
C5—C6	1.380 (4)	C14—H14	0.9300
			
C12—O3—H3A	111 (2)	C6—C7—N1	122.4 (3)
C8—N1—C7	127.0 (3)	C2—C7—N1	116.1 (2)
C8—N1—H1	122.7 (16)	N1—C8—C9	123.9 (3)
C7—N1—H1	109.9 (16)	N1—C8—H8	118.0
O2—C1—O1	124.2 (3)	C9—C8—H8	118.0
O2—C1—C2	119.2 (3)	C10—C9—C14	118.2 (3)
O1—C1—C2	116.6 (3)	C10—C9—C8	118.6 (3)
C3—C2—C7	117.6 (3)	C14—C9—C8	123.2 (3)
C3—C2—C1	117.9 (3)	C11—C10—C9	121.2 (3)
C7—C2—C1	124.5 (2)	C11—C10—H10	119.4
C4—C3—C2	121.4 (3)	C9—C10—H10	119.4
C4—C3—H3	119.3	C10—C11—C12	120.1 (3)
C2—C3—H3	119.3	C10—C11—H11	120.0
C5—C4—C3	119.9 (3)	C12—C11—H11	120.0
C5—C4—H4	120.0	O3—C12—C11	117.9 (3)
C3—C4—H4	120.0	O3—C12—C13	122.9 (3)
C4—C5—C6	120.5 (3)	C11—C12—C13	119.2 (3)
C4—C5—H5	119.8	C14—C13—C12	120.5 (3)
C6—C5—H5	119.8	C14—C13—H13	119.8
C5—C6—C7	119.2 (3)	C12—C13—H13	119.8
C5—C6—H6	120.4	C13—C14—C9	120.8 (3)
C7—C6—H6	120.4	C13—C14—H14	119.6
C6—C7—C2	121.4 (3)	C9—C14—H14	119.6
			
O2—C1—C2—C3	1.9 (4)	C8—N1—C7—C6	−11.1 (5)
O1—C1—C2—C3	−178.8 (3)	C8—N1—C7—C2	169.5 (3)
O2—C1—C2—C7	−177.6 (3)	C7—N1—C8—C9	179.4 (3)
O1—C1—C2—C7	1.7 (4)	N1—C8—C9—C10	172.3 (3)
C7—C2—C3—C4	0.6 (4)	N1—C8—C9—C14	−8.1 (5)
C1—C2—C3—C4	−179.0 (3)	C14—C9—C10—C11	1.9 (4)
C2—C3—C4—C5	−0.6 (5)	C8—C9—C10—C11	−178.6 (3)
C3—C4—C5—C6	0.1 (5)	C9—C10—C11—C12	0.0 (5)
C4—C5—C6—C7	0.4 (5)	C10—C11—C12—O3	178.3 (3)
C5—C6—C7—C2	−0.4 (4)	C10—C11—C12—C13	−2.1 (4)
C5—C6—C7—N1	−179.7 (3)	O3—C12—C13—C14	−178.0 (3)
C3—C2—C7—C6	0.0 (4)	C11—C12—C13—C14	2.3 (4)
C1—C2—C7—C6	179.5 (3)	C12—C13—C14—C9	−0.5 (4)
C3—C2—C7—N1	179.3 (3)	C10—C9—C14—C13	−1.6 (4)
C1—C2—C7—N1	−1.2 (4)	C8—C9—C14—C13	178.8 (3)

### Hydrogen-bond geometry (Å, º)

**Table:**

*D*—H···*A*	*D*—H	H···*A*	*D*···*A*	*D*—H···*A*
O3—H3*A*···O1^i^	0.95 (4)	1.71 (4)	2.656 (3)	173 (3)
N1—H1···O1	1.00 (3)	1.62 (3)	2.522 (3)	148 (2)
C6—H6···O2^ii^	0.93	2.51	3.397 (4)	160

Symmetry codes: (i) −*x*+1, −*y*, −*z*+1; (ii) −*x*−1/2, *y*+1/2, −*z*+1/2.
