# Supplementary figures and images for: Crystal structure of (E)-2-[(4-hy­droxy­benzyl­idene)aza­nium­yl]benzoate
Source: Acta Crystallogr Sect E Struct Rep Online. 2014 Aug 16;70(Pt 9):o1008. doi: 10.1107/S1600536814018273 (PMC4186171; doi:10.1107/S1600536814018273)

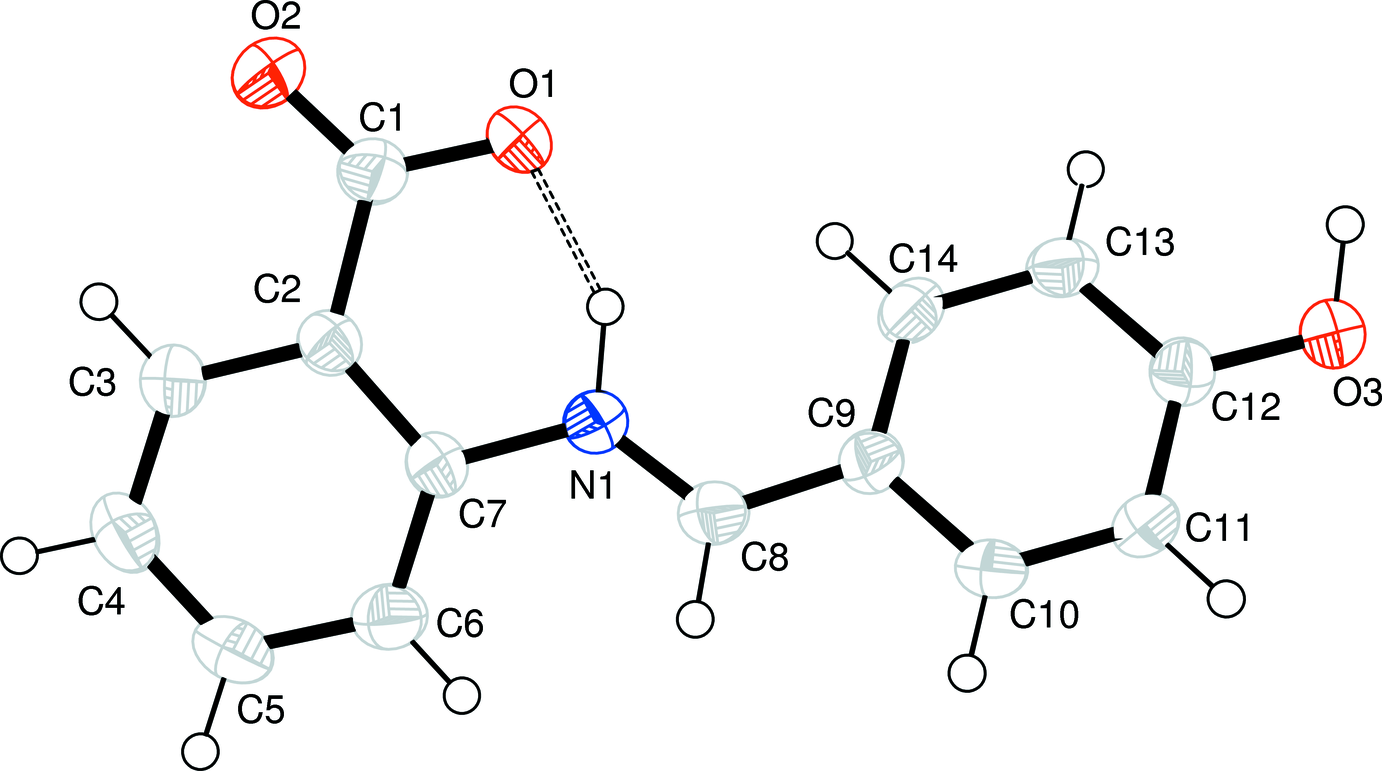

Supplement: Supplementary file 4 [file e-70-o1008-fig1.tif]

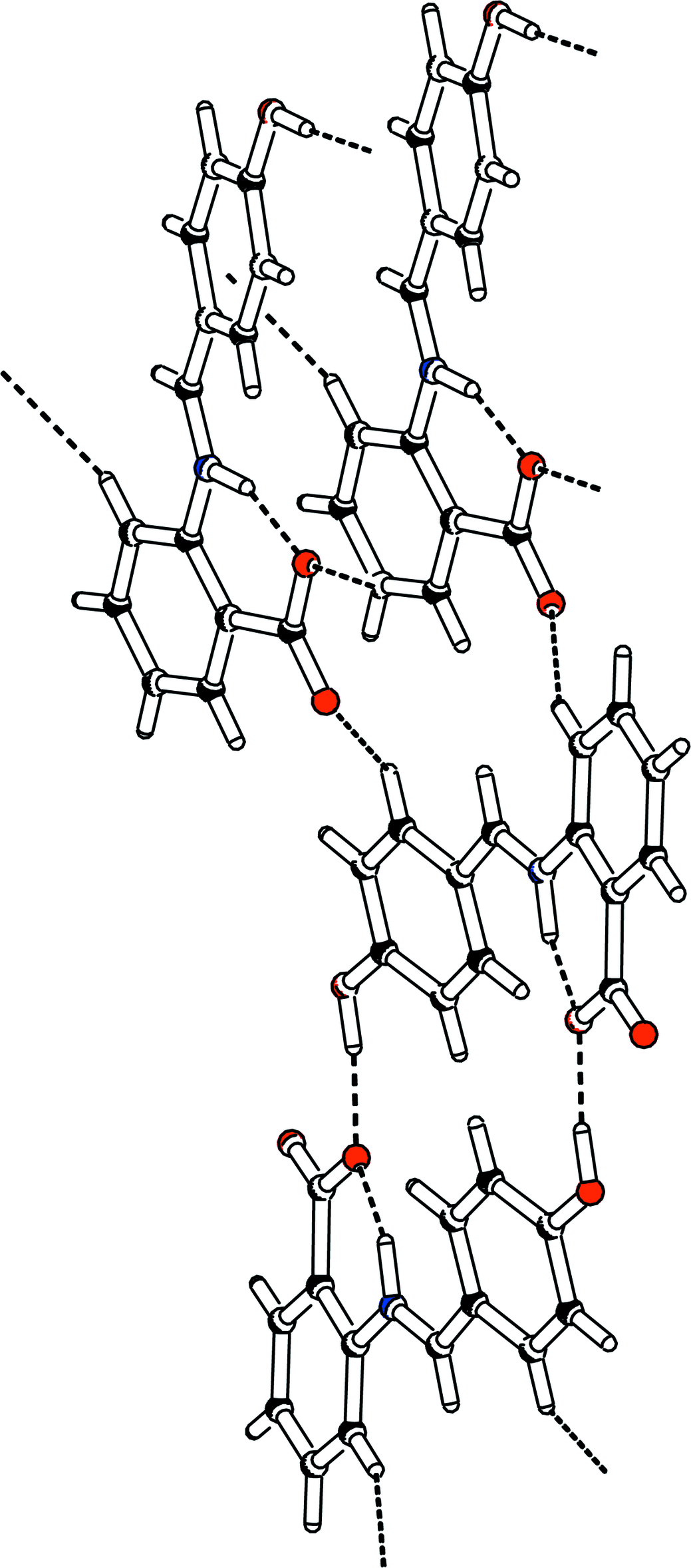

Supplement: Supplementary file 5 [file e-70-o1008-fig2.tif]
